# Deciphering the Irregular Risk of Stroke Increased by Obesity Classes: A Stratified Mendelian Randomization Study

**DOI:** 10.3389/fendo.2021.750999

**Published:** 2021-12-01

**Authors:** Xuelun Zou, Leiyun Wang, Linxiao Xiao, Zihao Xu, Tianxing Yao, Minxue Shen, Yi Zeng, Le Zhang

**Affiliations:** ^1^ Department of Neurology, Xiangya Hospital, Central South University, Changsha, China; ^2^ Department of Clinical Pharmacology, Xiangya Hospital, Central South University, Changsha, China; ^3^ Institute of Clinical Pharmacology, Central South University, Hunan Key Laboratory of Pharmacogenetics, Changsha, China; ^4^ Department of Spine Surgery and Orthopaedics, Xiangya Hospital, Central South University, Changsha, China; ^5^ Department of Epidemiology and Health Statistics, Xiangya School of Public Health, Central South University, Changsha, China; ^6^ Department of Geriatrics, Second Xiangya Hospital, Central South University, Changsha, China

**Keywords:** stroke, obesity, Mendelian randomization, body mass index, genome-wide association study

## Abstract

**Background:**

To investigate the relationship between different classes of obesity and stroke, we conducted a stratified Mendelian randomization (MR) study.

**Methods:**

The body mass index (BMI) data of 263,407 Europeans with three classes of obesity (obesity class I, 30 kg/m^2^ ≤ BMI < 35 kg/m^2^; obesity class II, 35 kg/m^2^ ≤ BMI < 40 kg/m^2^; obesity class III, 40 kg/m^2^ ≤ BMI) were extracted from the Genetic Investigation of ANthropometric Traits (GIANT) consortium. Summary-level data of stroke and its subtypes [ischemic stroke (IS) and intracerebral hemorrhage (ICH)] were obtained from the genome-wide association study (GWAS) meta-analysis, which was performed by the MEGASTROKE consortium. MR methods were used to identify the causal relationships.

**Results:**

The MR analysis revealed that both obesity class I [odds ratio (OR) = 1.08, 95% CI: 1.05–1.12, *p* = 1.0 × 10^-5^] and obesity class II (OR = 1.06, 95% CI: 1.03–1.09, *p* = 1 × 10^-4^) were significantly positively related to IS, while obesity class III was not (OR = 1.01, 95% CI: 0.96–1.06, *p* = 0.65). In contrast to IS, there was no class of obesity associated with ICH risk. Further examination of the relationship between obesity classification and IS subtypes revealed that certain degrees of obesity were related to large artery stroke (LAS) (OR = 1.14, 95% CI: 1.04–1.24, *p* = 2.8 × 10^-3^ for class I; OR = 1.08, 95% CI: 1.01–1.16, *p* = 0.002 for class II) and cardioembolic stroke (CES) (OR = 1.11, 95% CI: 1.02–1.20, *p* = 0.02 for class I; OR = 1.08, 95% CI: 1.02–1.15, *p* = 0.007 for class II).

**Conclusions:**

A higher risk of IS, but not ICH, could be linked to obesity classes I and II. A strong association between LAS and CES and obesity was observed among all IS subtypes in the obese population.

## Introduction

Stroke, the second leading cause of death and disability worldwide, has put huge economic pressure and healthcare burden on patients worldwide (1, 2). In 2017, there were about 6 million deaths due to stroke and a loss of 132.1 million stroke-related disability-adjusted life years (95% CI: 126.5–137.4) (2). A growing number of studies have confirmed the correlation between stroke and modifiable or non-modifiable risk factors such as smoking, drinking, hypertension, diabetes, and genetics (3). There is no doubt that the prevention and control of these risk factors will reduce the disease burden of stroke.

Obesity caused by abnormal metabolism, as one of the major health threats to all populations, is also viewed as a potential risk factor for stroke (4). As reported by the World Health Organization (WHO), approximately 1.9 billion adults are obese or overweight worldwide (5), and 3 million people died of obesity-related diseases in 2018 (6). Therefore, a large part of the disease burden of stroke may arise from obese people. Conventional observational studies may be affected by many confounders when investigating the causal relationship between exposure factors (obesity) and outcome effects (stroke). Factors such as the distribution of fat and the condition of health are difficult to control using typical analytic methods.

These shortcomings can be eliminated using Mendelian randomization (MR) studies. MR studies as nature’s randomized controlled trials are widely applied to assess the causality between exposure factors and outcome diseases (7). The MR method utilizes genetic variations, namely, single-nucleotide polymorphisms (SNPs), as mediating variables, which are objective factors free of influence from various confounding factors (8, 9). In MR studies, SNPs are used to determine whether an observational relationship between an exposure factor and an outcome disease is aligned with a causal effect (9). Therefore, MR studies have outstanding advantages in exploring the causal relationship between exposure factors and outcome diseases.

Previous studies have shown that obesity can lead to the accumulation of many human body adipose tissues, and adipose tissue can release a large number of inflammatory cytokines and anti-inflammatory factors, cytokines, and other factors (10–13). It also leads to a lack of oxygen to the body, oxidative stress, and chronic inflammation, causing blood vessel damage and extracellular matrix remodeling, such as pathological changes in vascular fibrosis (10, 13–16). All of these are potential stroke triggers that can accelerate brain vessel damage (17, 18). Observational studies have reported that obesity is associated with stroke, but the causality between them remains controversial (19, 20). Furthermore, in a previous MR study, abdominal adiposity has been shown to cause pathological processes in cerebrovascular diseases (21). Moreover, central adiposity has been reported to increase the risk of stroke (22). However, three other MR analyses (21–23) indicated that the BMI of obesity may not be related to stroke and its subtypes. This discrepancy may hinder the discovery of the inner links between obesity and stroke, which may be due to the irregular physiological status, different classes of obesity, or various subtypes of stroke. As reported in a genome-wide association meta-analysis study, different obesity classes presented obvious differences in genetics (24). Therefore, we speculated that only some obese people are at a higher risk of stroke, which may be due to genetic differences in their obesity classification. To confirm this assumption, we performed a stratified MR study to explore the causality between the classes of obesity and the risk of stroke.

Since the causal relationship between different obesity classes and risk of stroke with its subtypes is unclear, we conducted this stratified MR study to study the causal relationship.

## Methods

### Summary of Genome-Wide Association Study Data

The information on obesity exposure factors was acquired from the Genetic Investigation of ANthropometric Traits (GIANT) consortium (24), which is a publicly available database taken from the magnanimous data of genome-wide association study (GWAS). In detail, 263 and 407 Europeans were included to study the genetic factors associated with obesity (24). Obesity was divided into obesity class I (30 kg/m^2^ ≤ BMI < 35 kg/m^2^; 32,858 cases and 65,839 controls), obesity class II (35 kg/m^2^ ≤ BMI < 40 kg/m^2^; 9,889 cases and 62,657 controls), and obesity class III (40 kg/m^2^ ≤ BMI; 2,896 cases and 47,468 controls) (24).

The data on stroke and its subtypes in this study were acquired from the MEGASTROKE consortium (25) and the Common Metabolic Diseases Knowledge Portal (26). The summary statistics data of stroke were obtained from 446,696 subjects (40,585 cases and 406,111 controls). Among them, patients were ranked as having ischemic stroke (IS) (34,217 cases), large artery stroke (LAS) (4,373 cases), cardioembolic stroke (CES) (7,193 cases), and small vessel stroke (SVS) (5,386 cases) (25). Another common subtype of stroke is ICH, which was collected from another GWAS meta-analysis of 3,026 Europeans (about 1,545 cases and 1,481 controls) (26). Considering the different locations of hemorrhage, ICH was subtyped into lobar ICH (LICH, 686 cases) and non-lobar ICH (NLICH, 909 cases). Since this study was a retrospective study based on the public GWAS database as the published source, ethics approval was not required.

### Instrumental Single-Nucleotide Polymorphism Inclusion Criteria

SNPs related to obesity were selected from a meta-analysis of GWAS data. Information including age, sex, height, hip circumference, waist–hip ratio, and health condition of these obese patients was also acquired. Different SNPs related to the obesity classes were selected to evaluate the causal relationship between obesity and stroke (including IS and ICH). We also relaxed the selection criteria to *p* < 1 × 10^-6^ to retain enough SNPs, which could be included for downstream studies and make our results more reliable, as reported in a previous study (7). The parameters r^2^ = 0.01 and kb = 10,000 were chosen to remove the SNPs that could not pass the linkage disequilibrium test.

### Stratified Mendelian Randomization Analysis

Three R platform-supported packages, Mendelian randomization, TwoSampleMR (27), and Mendelian Randomization Pleiotropy RESidual Sum and Outlier (MR-PRESSO) (28), were utilized in this study. All analyses were completed using R (version 4.0.3) and R studio software.

The inverse-variance weighted (IVW) method was chosen as the primary method for analysis (29), which can obtain an accurate and stable estimate when the directional pleiotropy is of poor statistical significance (30). MR-Egger regression (31), weighted median estimation (WME) (32), and other robust MR methods were also used to show the causal association between each variable. MR-Egger regression methods can provide a robust estimate of the causal relationship when some instrumental variables violate the supposed selection criteria (31, 33). When the intercept term is close to zero, directional pleiotropy does not exist, and the results of the MR-Egger regression method will verge on IVW. WME has an advantage in controlling the Type 1 error rates of finite samples and has a stable estimator, although the invalid instrumental variables include values up to 50% (32).

In addition, to ensure the reliability and effectiveness of the MR study, the heterogeneity test, which can inspect the differences in all kinds of SNPs using Cochran’s Q statistics, was utilized to test the existence of heterogeneity (34). If there was heterogeneity in the larger sample, we needed to eliminate the SNPs that had small *p*-values by utilizing a random-effects model to assess the effect of MR. Therefore, we conducted a random-effects model to analyze the association between obesity class II and SVS, while the rest of the analyses adopted a fixed-effects model in this stratified MR analysis. Pleiotropy tests were performed in two ways: the intercept term of the MR-Egger regression method and the global and distortion test in the MR-PRESSO method (28). MR-PRESSO global and distortion tests, which could delete the SNPs gradually, were utilized to determine the presence or absence of abnormal values and to determine how to handle these accordingly (28). The leave-one-out sensitivity test was used to appraise whether the MR results were sensitized to single SNP changes (35). All of these parameters for model selection are listed in **Table 3**.

### Assumptions of the Mendelian Randomization Study

**Figure 1:**
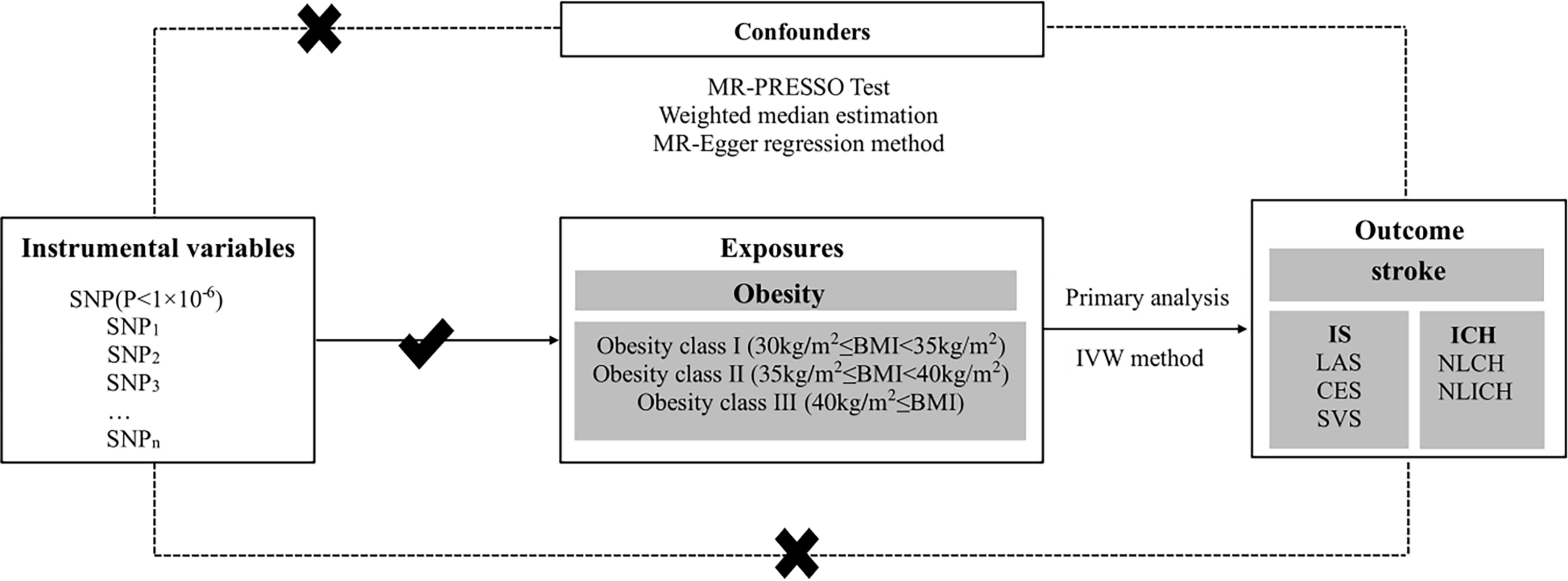
Conceptual framework figure of exploring the causal relationship in different obesity classes and stroke with its subtypes in the MR analysis. MR, Mendelian randomization; IS, ischemic stroke; LAS, large artery stroke; CES, cardioembolic stroke; SVS, small vessel stroke; ICH, intracerebral hemorrhage; LICH, lobar intracerebral hemorrhage; NLICH, non-lobar intracerebral hemorrhage; IVW, inverse-variance weighted; WME, weighted median estimation; MR-PRESSO, MR pleiotropy residual sum and outlier.

Three core assumptions must be satisfied to obtain valid results from MR studies (36, 37). 1) Instrumental variables (SNPs) must not correlate with confounders. To estimate this assumption, we checked each instrument variable in phewascatalog.org (38), where no statistical significance (*p* < 1 × 10^-6^) of association was detected. In addition, we carefully checked the exposure factors in the GWAS Catalog database and found no correlation with previously reported confounding factors (*p* < 1 × 10^-6^) (39). We defined smoking, unhealthy diet, physical activity, and sedentary time as associated confounding factors (40–42). 2) The direct association between instrumental variables (SNPs) and exposure (obesity) must be reliable. As shown in **Supplementary Tables S1–S3**, we found that all included SNPs were significantly related to exposure (obesity). To assess the pleiotropy of the selected SNPs, F statistics was used (43). When F ≤ 10, the selected SNPs were viewed as weak instrument biases, and thus they could not explain the observed exposure associations well (44). The F values of the instrumental variables selected based on the exposure variable ranged from 22 to 306, the details of which can be found in **Supplementary Tables S1–S3**. 3) Instrumental variables (SNPs) should affect the outcome (stroke) only by exposure (obesity), and there should be no other pathways. In order to eliminate the impact of other potential paths, we conducted the MR-Egger regression method and MR-PRESSO global test and found no existence of pleiotropy. The influence of potential pathways was also excluded, as presented in the *Results* section below (**Table 3**) (36). All assumptions are shown in **Figure 1**.

### Statistical Analyses

The statistical significance of the *p*-value in the multiple comparisons was defined as 0.0056, contributing to a Bonferroni correction of nine tests in the primary analysis. Three exposures (obesity class I, obesity class II, and obesity class III) and three main outcomes (stroke, IS, and ICH) were counted. Since the total causal relationship was confirmed in the primary analysis and was only used for exploratory analyses, the subtype analysis was performed without conducting *p*-value corrections (45).

## Results

In our study, MR analysis was used to explore the causal relationship between different classes of obesity and stroke with its subtypes, such as IS and ICH, as shown in **Figure 1**. SNPs were selected as instrumental variables, and different classes of obesity were viewed as exposure factors, while stroke and its subtypes were regarded as the outcome diseases in this study. Data for this study were derived from the different classes of obesity GWAS including 263,407 European populations and a 446,696 European stroke population.

### Selected Single-Nucleotide Polymorphisms

The instrumental variable SNPs between the different classes of obesity and the risk of IS subtypes were screened (**Supplementary Tables S1–S3**). As a result, 29 SNPs, 21 SNPs, and five SNPs passed the screening rules for obesity class I, obesity class II, and obesity class III, respectively. Among them, rs13130484 was not detected in the GWAS of CES. Therefore, this variation was excluded from the analysis of the causal relationship between obesity classes I and II and CES. The instrumental variables of SNPs that matched ICH were selected from the obesity data. Consequently, there were 24 SNPs found for obesity class I, 19 SNPs for obesity class II, and four SNPs for obesity class III. In obesity class I with risk of ICH, rs1514177, rs17203351, rs523288, rs8028313, and rs9816226 were removed because they were palindromic with intermediate allele frequencies, and rs7531118 was not detected in the GWAS of ICH. rs7538571 was removed for intermediate allele frequencies in obesity class II and ICH. In obesity class III with a risk of ICH, rs13104545 was not detected in the GWAS of ICH.

### Obesity Classes I and II but Not III Were Significantly Associated With the Risk of Ischemic Stroke

The IVW method was utilized for stratified MR analysis to assess the association between obesity and stroke. We found that the SNPs of obesity class I (OR = 1.08, 95% CI: 1.05–1.12, *p* = 7.4 × 10^-6^) and obesity class II (OR = 1.05, 95% CI: 1.02–1.08, *p* = 7 × 10^-4^) significantly increased the risk of stroke but not obesity class III (OR = 1.00, 95% CI: 0.94–1.06, *p* = 0.96). To determine the association between obesity classes and IS, which is one of the two common subtypes of stroke, we performed stratified MR analysis and found that genetic predispositions to obesity class I (OR = 1.08, 95% CI: 1.05–1.12, *p* = 1.0 × 10^-5^) and obesity class II (OR = 1.06, 95% CI: 1.03–1.09, *p* = 1 × 10^-4^) were positively associated with the risk of IS. However, no statistically significant causal relationship between obesity class III and the risk of IS could be established (OR = 1.01, 95% CI: 0.96–1.06, *p* = 0.65). These results are shown in **Table 1**. The power values of the relationship between stroke and obesity classes I, II, and III were 0.88, 0.66, and 0.82, respectively, which indicates that our negative observation for obesity class III is reliable.

**Table 1:** MR stratification analysis of obesity classes I, II, and III with the risk of IS and IS subtypes using the IVW method.

		Obesity class I (n = 29)		Obesity class II (n = 21)		Obesity class III (n = 5)	
		OR (95% CI)	*p* value	OR (95%CI)	*p* value	OR (95% CI)	*p* value
**Stroke**		1.08 (1.05–1.12)	**7.4 × 10^-6^**	1.05 (1.02–1.08)	**7 × 10^-4^**	1.00 (0.94–1.06)	0.96
**IS**		1.08 (1.05–1.12)	**1.0 × 10^-5^**	1.06 (1.03–1.09)	**1 × 10^-4^**	1.01 (0.96–1.06)	0.65
	LAS	1.14 (1.04–1.24)	**2.8 × 10^-3^**	1.08 (1.01–1.16)	**0.002**	1.04 (0.80–1.36)	0.75
	CES	1.11 (1.02–1.20)	**0.02**	1.08 (1.02–1.15)	**0.007**	1.02 (0.93–1.12)	0.64
	SVS	1.07 (0.97–1.17)	0.17	1.03 (0.95–1.13)	0.42	1.07 (0.98–1.17)	0.12

IS, ischemic stroke; LAS, large artery stroke; CES, cardioembolic stroke; SVS, small vessel stroke; IVW, inverse-variance weighted method.

Power _obesity class I_ = 0.88, Power _obesity class II_ = 0.66, Power _obesity class III_ = 0.82. All values which are lower than 0.05 are marked in the bold font, indicating that they are statistically significant.

In the stratified MR analysis for all three IS subtypes (LAS, CES, and SVS), we found that obesity class I (OR = 1.14, 95% CI: 1.04–1.24, *p* = 2.8 × 10^-3^) and obesity class II (OR = 1.08, 95% CI: 1.01–1.16, *p* = 0.002) could increase the risk of LAS but not obesity class III (OR = 1.04, 95% CI: 0.80–1.36, *p* = 0.75). The risk of CES was increased by obesity class I (OR = 1.11, 95% CI: 1.02–1.20, *p* = 0.02) and obesity class II (OR = 1.08, 95% CI: 1.02–1.15, *p* = 0.007) but was not related to obesity class III (OR = 1.02, 95% CI: 0.93–1.12, *p* = 0.64). Interestingly, obesity class I (OR = 1.07, 95% CI: 0.97–1.17, *p* = 0.17), obesity class II (OR = 1.03, 95% CI: 0.95–1.13, *p* = 0.42), and obesity class III (OR = 1.07, 95% CI: 0.98–1.17, *p* = 0.12) presented no causal relationship with SVS. The intercept from the MR-Egger regression (heterogeneity test) and Cochran’s Q statistics (pleiotropy test) did not show statistical significance between obesity class I, obesity class II, and obesity class III and the risk of stroke, IS, and other IS subtypes (**Table 3**).

### Obesity Was Not Associated With Intracerebral Hemorrhage and Its Subtypes

As **Table 2** shows, no causal relationship between obesity class I (OR = 1.05, 95% CI: 0.80–1.37, *p* = 0.75), obesity class II (OR = 1.07, 95% CI: 0.88–1.32, *p* = 0.49), and obesity class III (OR = 0.93, 95% CI: 0.76–1.11, *p* = 0.43) with a risk of ICH was found through the IVW analysis. Moreover, obesity class I (OR = 1.10, 95% CI: 0.81–1.51, *p* = 0.53), obesity class II (OR = 1.01, 95% CI: 0.80–1.28, *p* = 0.94), and obesity class III (OR = 0.95, 95% CI: 0.68–1.30, *p* = 0.74) were not related to the ICH subtype of LICH. Obesity class I (OR = 1.06, 95% CI: 0.78–1.45, *p* = 0.70), obesity class II (OR = 1.19, 95% CI: 0.91–1.55, *p* = 0.21), and obesity class III (OR = 0.98, 95% CI: 0.88–1.09, *p* = 0.89) were not associated with NLICH. No heterogeneity and pleiotropy could be detected between obesity classes I, II, and III and the risk of ICH and its subtypes, which indicated the reliability of our analysis (**Table 3**).

**Table 2:** MR stratification analysis of obesity classes I, II, and III with the risk of ICH and ICH subtypes using the IVW method.

		Obesity class I (n = 24)		Obesity class II (n = 19)		Obesity class III (n = 4)	
		OR (95% CI)	*p* value	OR (95% CI)	*p* value	OR (95% CI)	*p* value
**Stroke**		1.08 (1.05–1.12)	**7.4 × 10^-6^**	1.05 (1.02–1.08)	**7 × 10^-4^**	1.00 (0.94–1.06)	0.96
**ICH**		1.05 (0.80–1.37)	0.75	1.07 (0.88–1.32)	0.49	0.96 (0.76–1.31)	0.81
	LICH	1.10 (0.81–1.51)	0.53	1.01 (0.80–1.28)	0.94	0.96 (0.73–1.25)	0.74
	NLICH	1.06 (0.78–1.45)	0.70	1.19 (0.91–1.55)	0.21	1.02 (0.72–1.46)	0.89

ICH, intracerebral hemorrhage; LICH, lobar intracerebral hemorrhage; NLICH, non-lobarintracerebral hemorrhage; IVW, inverse-variance weighted method. All values which are lower than 0.05 are marked in the bold font, indicating that they are statistically significant.

**Table 3:** The outcome of the sensitivity analysis of obesity with the risk of stroke.

Method		Cochran’s Q	HT (*p*)	intercept	PT (*p*)	GT (RSSobs)	GT (*p*)
**Obesity class I**	**Stroke**	20.7	0.84	-0.0001	0.97	24.9	0.77
	**IS**	26.5	0.55	-0.0017	0.74	30.8	0.49
	LAS	23.2	0.72	-0.0080	0.49	24.9	0.77
	CES	35.5	0.13	0.0112	0.35	40.6	0.11
	SVS	35.7	0.10	-0.0103	0.39	39.9	0.11
	**ICH**	31.1	0.12	-0.0029	0.94	36.2	0.23
	LICH	24.3	0.39	-0.0094	0.82	29.1	0.52
	NLICH	28.7	0.19	0.0034	0.93	33.6	0.30
**Obesity class II**	**Stroke**	23.2	0.28	0.0035	0.58	24.8	0.32
	**IS**	23.0	0.29	0.0040	0.55	24.7	0.32
	LAS	17.8	0.60	-0.0012	0.94	19.2	0.63
	CES	16.8	0.66	0.0148	0.25	19.3	0.64
	SVS	39.5	0.00	-0.0020	0.91	43.5	0.00
	**ICH**	21.2	0.15	0.0540	0.23	29.9	0.13
	LICH	15.3	0.57	0.0317	0.54	17.9	0.59
	NLICH	29.4	0.05	0.0828	0.16	35.2	0.04
**Obesity class III**	**Stroke**	6.39	0.17	-0.0220	0.37	8.71	0.24
	**IS**	4.21	0.37	-0.0160	0.46	5.81	0.49
	LAS	8.52	0.07	-0.0890	0.18	11.9	0.14
	CES	1.79	0.77	0.0388	0.37	3.67	0.69
	SVS	4.82	0.31	-0.0235	0.63	7.21	0.34
	**ICH**	6.75	0.08	-0.1036	0.64	9.57	0.17
	LICH	2.13	0.54	-0.0699	0.67	3.03	0.64
	NLICH	6.38	0.09	-0.0770	0.76	9.14	0.20

IS, ischemic stroke; LAS, large artery stroke; CES, cardioembolic stroke; SVS, small vessel stroke; ICH, intracerebral hemorrhage; LICH, lobar intracerebral hemorrhage; NLICH, non-lobar intracerebral hemorrhage; SNP, single-nucleotide polymorphism; IVW, inverse-variance weighted; WME, weighted median estimation; MR-PRESSO, MR pleiotropy residual sum and outlier; HT, heterogeneity test; PT, pleiotropy test; GT, MR-PRESSO global test.

### Validation of the Associations Using Various Stratified Mendelian Randomization Methods

**Figure 2:**
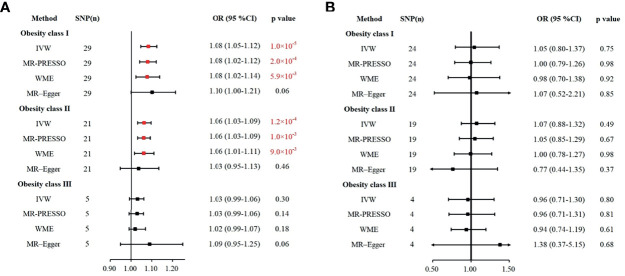
Various methods to assess the impacts of obesity class I, obesity class II, and obesity class III on IS **(A)** and ICH **(B)**. **(A)** The causal effect of obesity and risk of IS. **(B)** The causal effect of obesity and the risk of ICH. IS, ischemic stroke; ICH, intracerebral hemorrhage; IVW, inverse-variance weighted; WME, weighted median estimation; MR-PRESSO, MR pleiotropy residual sum and outlier.

To ensure the stability and reliability of the results, we used three other methods, MR-PRESSO, WME, and MR-Egger, to test whether the classes of obesity were associated with the risk of IS and ICH. As expected, obesity class I had a causal relationship with the risk of IS in the MR-PRESSO (OR = 1.08, 95% CI: 1.02–1.12, *p* = 2.0 × 10^-4^) and WME (OR = 1.08, 95% CI: 1.02–1.14, *p* = 5.9 × 10^-3^) analyses. Similarly, the MR-PRESSO (OR = 1.06, 95% CI: 1.03–1.09, *p* = 1.0 × 10^-3^) and WME (OR = 1.06, 95% CI: 1.01–1.11, *p* = 9.0 × 10^-3^) analyses confirmed the causal effect of obesity class II on the risk of IS. Obesity class III showed no association with the risk of IS in the MR-PRESSO, WME, and MR-Egger analyses, which is in agreement with our results calculated using the IVW method. Likewise, obesity classes I, II, and III were not correlated with ICH in the MP-PRESSO, WEM, or MR-Egger analyses. All these results were consistent with the IVW analyses in the previous section and are summarized in **Figure 2**.

### Single-Nucleotide Polymorphisms Affect the Causal Relationship Between Obesity Class I/Class II and Ischemic Stroke/Cardioembolic Stroke

**Figure 3:**
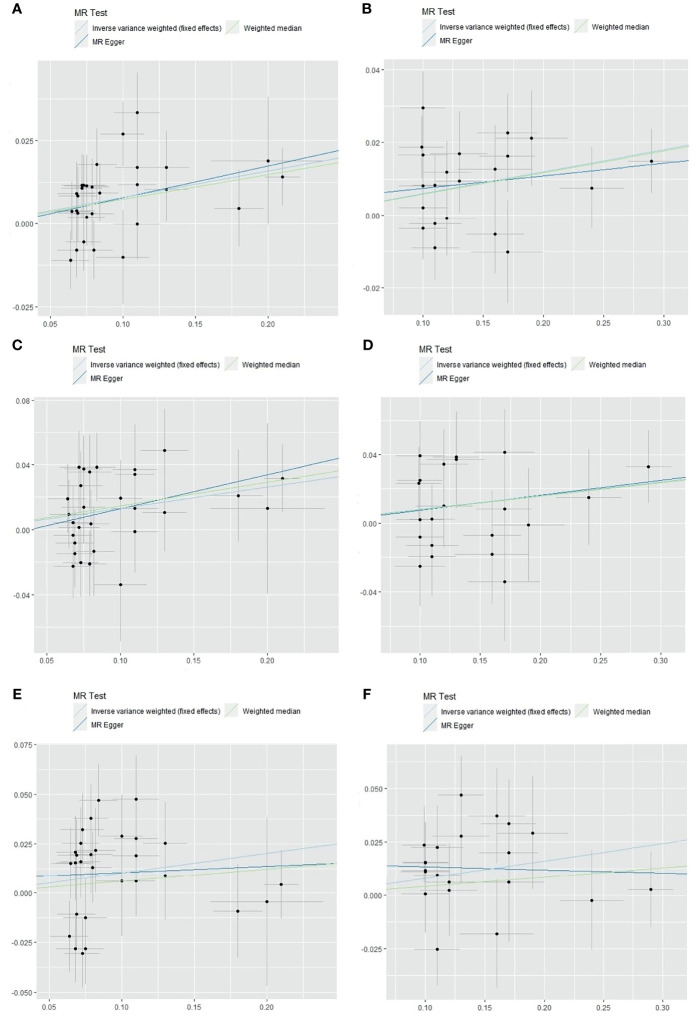
The scatter plot of obesity classes I and II with the risk of IS, LAS, and CES in the MR analysis. **(A)** Analysis of obesity class I and risk of IS. **(B)** Analysis of obesity class II and risk of IS. **(C)** Analysis of obesity class I and risk of LAS. **(D)** Analysis of obesity class II and risk of LAS. **(E)** Analysis of obesity class I and risk of CES. **(F)** Analysis of obesity class II and risk of CES. IS, ischemic stroke; LAS, large artery stroke; CES, cardioembolic stroke; MR, Mendelian randomization.

**Figure 4:**
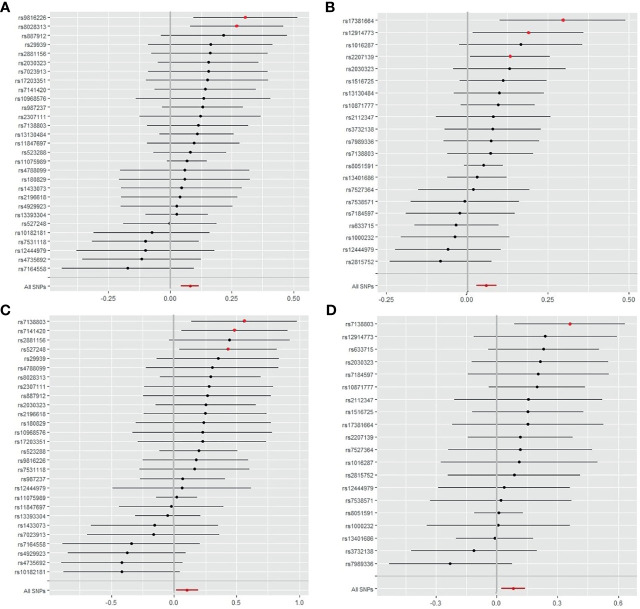
Forest plot of SNPs of obesity classes I and II correlated with the risk of IS and CES. **(A)** The single effect of the SNPs in obesity class I and the risk of IS. **(B)** Single effects of the SNPs on obesity class II and the risk of IS. **(C)** Single effects of the SNPs on obesity class I and risk of CES. **(D)** Single effects of the SNPs on obesity class II and the risk of CES. IS, ischemic stroke; CES, cardioembolic stroke; SNP, single-nucleotide polymorphism.

As shown in **Figure 3**, IS, LAS, and CES were significantly associated with obesity classes I and II. As most MR studies reported, the SNPs that contributed to obesity class I and obesity class II also presented similar causality associations with IS and its subtypes (**Figure 4**). In the forest plot containing each SNP effect value, we found that all 29 SNPs were associated with the causal relationship between obesity class I and IS (**Figure 4A**). Among them, two SNPs (rs9816226 and rs8028313) of obesity class I may have contributed to the incidence of IS to a great degree. Similarly, three SNPs (rs17381664, rs12914773, rs2207139), which are the causes of obesity class II, can significantly increase the risk of IS, as shown in **Figure 4B**. Furthermore, we found that three SNPs (rs7138803, rs7141420, and rs527248) may be the potential reasons for why obesity class I can increase the risk of CES (**Figure 4C**), and one SNP (rs7138803) from obesity class II played an outstanding role in CES (**Figure 4D**).

In addition, no outliers could be gauged in the leave-one-out analysis between obesity and the risk of IS. In addition, no outliers were found between obesity classes I, II, or III and stroke and all subtypes in the MR-PRESSO global test (**Table 3**).

## Discussion

To the best of our knowledge, this is the first study to investigate the causal relationship between different obesity classes and the risk of stroke and its subtypes, particularly conducted using the epidemiology data of a large number of patients from GWAS meta-analysis findings. Our findings confirmed that obesity classes I and II were associated with IS. Among the subtypes of IS, obesity classes I and II significantly influenced LAS and CES. Although suggested by their genetic characteristics, the causal relationship between obesity class III and stroke was not observed in our MR study. Whether the relationship between obesity class III and stroke exists in other populations needs to be confirmed with further research. In addition, ICH and its subtypes did not reach statistical significance with any class of obesity in this stratified MR analysis.

As our study indicated, there was no causal relationship between obesity class III and stroke, which is consistent with previous studies (22, 23). However, our results confirmed a specific correlation between obesity class I, obesity class II, and the risk of stroke, which is different from the results of previous studies (22–24). Our study focused on the relationship between different subtypes of obesity and found that only a certain degree of obesity increased the risk of stroke. This is in line with the common knowledge that different degrees of obesity have different effects on the metabolism and blood vessels of the body. Moreover, previous GWASs on obesity also confirmed genetic differences in obesity classification (24). Therefore, our findings on the association between obesity and stroke are potentially related to the degrees of obesity studied. Further studies are needed to explore the mechanism between different degrees of obesity and the occurrence of IS.

Different IS subtypes have different pathogenesis mechanisms; therefore, we further explored the influence of different obesity classifications on IS subtypes. In the subtype analysis of IS, obesity likely plays a critical role in the mechanisms of morbidity in LAS, which can be attributed to the atherosclerosis of large and medium-sized vessels due to the destruction of the microenvironment induced by the deposition of cholesterol *via* a macrophage-sterol-responsive network triggered by obesity (46, 47). Subsequently, the incidence of atherosclerosis can increase susceptibility to ischemic events (48–50) and LAS. In addition, abnormal massive accumulation of adipocytes in obese patients results in increased secretion of leptin, adiponectin, and pro-inflammatory cytokines, which subsequently aggravate the progression of atherosclerosis and thrombosis of cardiac and cerebral vessels and accelerate the development of LAS (49, 51, 52).

An association between genetic predisposition to obesity class I and the risk of CES was revealed in this study through MR analysis. Obesity is one of the most important predictors of atrial fibrillation and is associated with a decrease in systemic vascular resistance and an increase in cardiac output (53). A continuous state of the supercycle is prone to left ventricular diastolic dysfunction and left artery embolism, which could trigger atrial fibrillation and thus cause CES (53–55). The influence of obesity on atrial fibrillation frequently depends on the degree and duration of obesity (53, 56). In contrast, our study provided no significant evidence for an association between obesity and SVS (all *p* > 0.05). Convincing evidence has shown that obese individuals are more susceptible to atherosclerosis and insulin resistance (48, 57), while the association between SVS and insulin resistance and atherosclerosis has not been established (58).

Interestingly, we also found that a higher degree of obesity was not associated with a higher risk of stroke at the genetic level. Our MR analysis indicated that a higher BMI was only associated with a higher risk of stroke within a certain BMI range (30 kg/m^2^ ≤ BMI < 40 kg/m^2^), which is in contrast to a previous study (59). A systematic review and meta-analysis of BMI and stroke also showed that the relationship between BMI and stroke has a J-type distribution. With a BMI >25 kg/m^2^, the risk of stroke rapidly increases (60). The difference between our findings and the above report could be attributed to the potential influence of mediating variables in stroke such as hypertension and smoking (61, 62). Another potential interpretation is that morbid obesity (BMI >40 kg/m^2^) leads to a decrease in endothelial cell shear stress, which reduces the risk of atherosclerosis due to increases in blood vessel diameters and reductions of injuries in vascular endothelial cells (49, 63). In addition, an animal model study on the obesity paradox indicated that extremely obese mice were deficient in microRNA-155 (64), which acts on the 3-terminal noncoding region of nitric oxide synthase in vascular endothelial cells and regulates the function of vascular endothelial cells (65). Deficiency in microRNA-155 reduces the risk of atherosclerosis by improving the generation rate of vascular endothelium (64, 65). Moreover, in morbidly obese people, some factors secreted by adipocytes may play different roles. Adiponectin is an exceptive adipokine that can reduce the risk of atherosclerosis in the morbidly obese population by regulating the inflammatory reaction of the endothelium, inhibiting the scavenger receptor of macrophages, and inhibiting the transformation of macrophages to foam cells (49, 66, 67). Finally, the risk of stroke is reduced by the low occurrence of atherosclerosis in the morbidly obese population.

Although our study found that obesity class III was not associated with genetic risk of stroke, some studies have shown that obesity disrupts the balance between pro-inflammatory adipokines and anti-inflammatory adipokines (10–13). The equilibrium is transformed into pro-inflammatory mediators, and pro-inflammatory factors such as leptin, resistin, chemerin, visfatin, retinol binding protein 4, and lipocalin 2 may increase sharply, leading to chronic inflammation of the blood vessels (10, 68, 69). Chronic vascular inflammation and immune cell infiltration can promote remodeling of the extracellular matrix and vascular fibrosis (16, 70, 71). Therefore, class III obesity does not protect against stroke. Obesity classes I and II are associated with stroke, but the effects of unhealthy lifestyle obesity on the risk of stroke should be avoided.

Our study found that no class of obesity was related to ICH and its subtypes, which is different from previous observational studies. These may imply the existence of confounding factors in observational studies that interfere with causality (72, 73). Although several observational studies have considered various adjustments for confounding factors, some unknown confounders may not have been found (74). Taken together with previous reports (72, 75), hypertension, hyperlipidemia, diabetes, and other factors, but not obesity, may be the direct causes of ICH. In addition, a previous MR study confirmed that hypertension plays a role in mediating causality in abdominal obesity-induced stroke (21). Moreover, we carefully compared the previously published GWAS data on hypertension with the data on obesity included in this study and found that the genetic loci were different (24, 76). This also confirms that there is no genetic association between obesity and ICH and that hypertension may be the main cause of ICH. All these confounders may contribute more to ICH than the SNPs analyzed in our MR study.

The obvious advantages of our study lie in the following points. First, this MR analysis is the first study to explore the relationship between different obesity grades and stroke and its subtypes in a large sample size using the MR method. We also tested the heterogeneity, directional pleiotropy, and outliers using a variety of sensitivity analysis methods to confirm our results. Second, this study uncovered the association of class I and class II but not class III obesity with stroke for the first time. Finally, we found that some SNP sites could be potential predictors of IS and CES in class I and class II obese populations. These SNPs may be clinically significant for the prevention and treatment of IS and CES in obese people in the future.

This study had some limitations. First, the population was derived from Europe. To our knowledge, ethnic and regional differences can influence the incidence and prevalence of stroke and obesity (77). Developing regions such as Asia and Africa have higher stroke burdens (1) and have higher incidence and fatality rates due to stroke (2). However, current GWAS data for stroke in Asians and Africans were limited, which hampers the downstream analyses for other populations. Therefore, our results should be generalized with caution to other populations that are not of European descent, and further GWASs and MR studies for other populations are warranted to determine the causal relationship between obesity and the risk of stroke. Second, we found that some of the SNPs in obesity classes I and II were positively associated with IS, especially CES. These SNPs and their impact on the predisposition to stroke warrant close attention for stroke prevention in the obese population in further research. In clinical practice, these SNPs may have great significance in the prevention of IS, especially CES, for obese people with a certain BMI distribution (30 kg/m^2^ ≤ BMI < 40 kg/m^2^). Third, some personalized features of obese patients were not available in this study. For example, information about the distribution of fat and the diet of these obese patients would be helpful if available. As some studies reported, the differences between general obesity and central obesity also showed different impacts on the stroke risks (22). This also confirms the contribution of different obesity subgroups like patients with different fat distributions and indicated that the importance of analyzing from different obesity perspectives can explore the effects of different obesity classes on stroke. Therefore, in the future, the inclusion of different personalized characteristics can be considered in obese patients to explore the causal relationship between obesity and stroke in more detail.

## Conclusion

In summary, we found that not all subtypes of stroke are associated with obesity, namely, ICH and its subtypes. Interestingly, different classes of obesity did not contribute equally to stroke, as obesity classes I and II were more likely to be the cause of stroke. Moreover, we found that some SNPs in class I and II obesity patients may be the possible culprits of IS, especially CES, in these populations. Future studies focusing on the function or intervention of these SNPs may provide a preventive solution for stroke in obese populations.

## Data Availability Statement

The original contributions presented in the study are included in the article/**Supplementary Material**. Further inquiries can be directed to the corresponding author.

## Author Contributions

LZ and XZ designed the research and determined the structure of the paper. XZ, LX, and LW selected the references and contributed to the writing. XZ, LX, LW, ZX, and TY helped to analyze the results of the study. YZ, MS, and LZ contributed to the revision and finalization of the article. All authors contributed to the article and approved the submitted version.

## Funding

This project was supported by the National Science and Technology Foundational Resource Investigation Program of China (Grant No. 2018FY100900) and the National Natural Science Foundation of China (Grant No. 2016JJ2164).

## Conflict of Interest

The authors declare that the research was conducted in the absence of any commercial or financial relationships that could be construed as a potential conflict of interest.

## Publisher’s Note

All claims expressed in this article are solely those of the authors and do not necessarily represent those of their affiliated organizations, or those of the publisher, the editors and the reviewers. Any product that may be evaluated in this article, or claim that may be made by its manufacturer, is not guaranteed or endorsed by the publisher.
